# Transmission of Specific Genotype Streptomycin Resistant Strains of *Mycobacterium tuberculosis *in the Tokyo Metropolitan Area in Japan

**DOI:** 10.1186/1471-2334-9-138

**Published:** 2009-08-26

**Authors:** Akihiro Ohkado, Yoshiro Murase, Masaaki Mori, Naoki Hasegawa, Goro Otsuka, Michiko Nagamine, Hideo Maeda, Kazuhiro Uchimura, Masako Ohmori, Norio Yamada, Shinji Maeda, Seiya Kato, Toru Mori, Nobukatsu Ishikawa

**Affiliations:** 1 Department of Epidemiology and Clinical Research, Research Institute of Tuberculosis, Japan Anti-Tuberculosis Association, Matsuyama 3-1-24, Kiyose, Tokyo, Japan; 2 Department of Reference for Mycobacteria, Research Institute of Tuberculosis, Japan Anti-Tuberculosis Association, Kiyose, Tokyo, Japan; 3 Health Centre, Keio University, Yokohama, Japan; 4 Department of Respiratory Medicine, Keio University, Yokohama, Japan; 5 Department of Health and Clinical Medicine, Bureau of Health and Welfare, Kawasaki, Japan; 6 Bureau of Social Welfare and Public Health, Tokyo Metropolitan Government, Shinjuku, Tokyo, Japan; 7 Department of International Cooperation, Research Institute of Tuberculosis, Japan Anti-Tuberculosis Association, Kiyose, Tokyo, Japan; 8 Research Institute of Tuberculosis, Japan Anti-Tuberculosis Association, Kiyose, Tokyo, Japan

## Abstract

**Background:**

From 2003 through to 2004, an outbreak of tuberculosis was identified at a university campus in Yokohama City, located in the southern part of the Tokyo Metropolitan Area (TMA). All *Mycobacterium tuberculosis *(*M. tuberculosis*) strains detected with regards to this outbreak turned out to be Streptomycin resistant with matched patterns of 14 IS*6110 *bands of Restriction Fragment Length Polymorphism (RFLP). The *M. tuberculosis *bacilli, which had the matched IS*6110 *band patterns with resistance to Streptomycin to those of bacilli isolated in the outbreak, were also concurrently detected through either the population-based or the hospital-based DNA fingerprinting surveillance of *M. tuberculosis *either in Shinjuku City or in Kawasaki City respectively.

The aim of the present study is to describe the spread of the specific genotype strains of *M. tuberculosis *in the TMA as observed in the above incident, and to identify the possible transmission routes of the strains among people living in urban settings in Japan.

**Methods:**

We applied Variable Numbers of Tandem Repeats (VNTR) analysis to all *M. tuberculosis *isolates which were resistant to Streptomycin with a matched IS*6110*-RFLP band pattern (M-strains). They were isolated either from cases related to the tuberculosis outbreak that happened at a university, or through DNA fingerprinting surveillance of *M. tuberculosis *both in Shinjuku City and in Kawasaki City. For VNTR analysis, 12MIRU loci, 4ETR loci, seven loci by Supply, four loci by Murase (QUB15, Mtub24, VNTR2372, VNTR3336) were selected.

**Results:**

Out of a total of 664 isolates collected during the study period, 46 isolates (6.9%) were identified as M-strains. There was a tendency that there was a higher proportion of those patients whose isolates belonged to M4-substrains, with four copies of tandem repeat at the ETR-C locus, to have visited some of the internet-cafés in the TMA than those whose isolates belonged to M5-substrains, with five copies at the ETR-C locus, although statistically not significant (38.1% vs. 10.0%, Exact p = 0.150).

**Conclusion:**

Although firm conclusions could not be reached through the present study, it suggested that we have to take into consideration that tuberculosis can be transmitted in congregated facilities like internet cafés where tuberculosis high-risk people and general people share common spaces.

## Background

A population-based DNA fingerprinting surveillance of *Mycobacterium tuberculosis *(*M. tuberculosis*) using IS*6110 *Restriction Fragment Length Polymorphism (IS*6110*-RFLP) analysis has been conducted since 2002 in Shinjuku City, located in the centre of the Tokyo Metropolitan Area (TMA) with approximately 300 000 residents, in order to evaluate the transmission status of *M. tuberculosis *in an urban setting in Japan [1]. In addition, since 2004 a hospital-based DNA fingerprinting surveillance of *M. tuberculosis *has been conducted at a public hospital in Kawasaki City, located in the south of the TMA with approximately 1.4 million residents [2]. At the same time, from 2003 through to 2004, an outbreak of tuberculosis (TB) was identified at a university campus in Yokohama City, located just south of Kawasaki City and with a population of approximately 3.7 million [3]. All *M. tuberculosis *strains detected with regards to this outbreak at the university campus turned out to be Streptomycin resistant and the IS*6110*-RFLP analysis showed matched patterns with 14 IS*6110 *bands. In addition *M. tuberculosis *bacilli, which had the matched IS*6110 *band patterns with resistance to Streptomycin to those of bacilli isolated in the outbreak, were also concurrently detected through either the population-based or the hospital-based DNA fingerprinting surveillance of *M. tuberculosis *either in Shinjuku City or in Kawasaki City respectively.

The aim of the present study is to describe the spread of the specific genotype strains of *M. tuberculosis *in the TMA as observed in the above incident, and to identify the possible transmission routes of the strains among people living in urban settings in Japan.

## Methods

**Figure 1:**
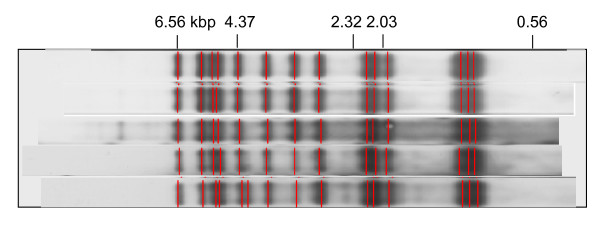
**Representative IS *6110*-RFLP Band Patterns of the M-strains including a pattern with one band difference**.

The design of the study is descriptive, to combine the epidemiological data of bacillary positive tuberculosis patients and DNA fingerprinting analysis results of *M. tuberculosis *isolated from each of the patients. We applied Variable Numbers of Tandem Repeats (VNTR) analysis to all *M. tuberculosis *isolates that were resistant to Streptomycin with a matched IS*6110*-RFLP band pattern (Figure 1). They were isolated either through the contact investigation of TB with regards to the TB outbreak that happened at a university campus in Yokohama City, from cases with possible links with the other outbreaks as suspected by public health centre (PHC) staff in the TMA, or through the population-based or hospital-based DNA fingerprinting surveillance of *M. tuberculosis *either in Shinjuku City or in Kawasaki City respectively. These two databases, which were independent of each other, were chosen because these were the only ones for us to investigate how these strains spread in the TMA. As the time of this study, there is no database covering the TMA in its entirety. In addition, the database of Kawasaki City, although its coverage among bacillary positive TB patients registered in the City was relatively low, approximately 21% (unpublished data), was chosen because we suspected there existed some link between the M-strains isolated from TB patients at the university campus in Yokohama City and TB bacilli in the Kawasaki City database for the university campus was located very close to the border between the two cities. The study period was set from September 2002 through to January 2008 because the outbreak incident at the university campus in Yokohama City, which prompted us to investigate the transmission situation of the M-strains in the TMA, seemed to have subsided by the end of 2006.

Drug susceptibility tests (DST) were performed at the laboratories where the strains of *M. tuberculosis *bacilli were initially isolated from the patients with TB. The DST results were collected by the PHC in the routine data collection process from doctors treating the patients. The DST was conducted with the proportion method with 1% Ogawa egg slant, with the critical concentration of drug at 0.2 mcg/ml for Isoniazid, 40 mcg/ml for Rifampicin, 10 mcg/ml for Streptomycin, and 2.5 mcg/ml for Ethambutol. The DST results were verified at the Research Institute of Tuberculosis (RIT), Tokyo, unless the laboratories where the DST was performed had been included in the network of quality assurance by RIT.

Once isolated at laboratories, cultures of *M. tuberculosis *from each patient were sent to RIT, for DNA fingerprinting analysis. The standardised method of DNA fingerprinting using IS*6110 *by the restriction fragment length polymorphism analysis was applied [4]. The 27-locus VNTR analysis was performed using primers designed specifically to each of the loci selected. In the current VNTR analysis, 12 loci by Mazars *et al*. i.e., MIRU 2, 4, 10, 16, 20, 23, 24, 26, 27, 31, 39, 40 [5]; four loci by Frothingham *et al*. i.e., ETR A, B, C, F [6]; supplemented with seven loci recommended by Supply *et al*. i.e., QUB11b, QUB26, Mtub04, Mtub21, Mtub30, Mtub39, VNTR4156 [7]; and the other four loci recommended by Murase *et al*. i.e., QUB15, Mtub24, VNTR2372, VNTR3336 [8] were selected. The last four loci were added in order to increase the discriminatory power in the context of high prevalence of Beijing-genotype *M. tuberculosis *strains in Japan [9,10]. The VNTR profile of each of the analyzed *M. tuberculosis *was described as a set of figures of tandem repeat copy number according to this loci set order as mentioned above. The VNTR analysis was made following the previously described methods using Ex Taq with GC PCR buffer I (Takara Bio) [8,11]. In brief, the PCR mixture was prepared in a 20 ml volume with 16GC PCR buffer I, 0.5 U Ex Taq, 200 mM each of four dNTPs, 0.5 mM each of the primer set and 10 ng template DNA. PCR was carried out for all loci under the following conditions: initial denaturation at 94 uC for 5 minutes, and then 35 cycles of 94 uC for 30 seconds, 63 uC for 30 seconds and 72 uC for 3 minutes, followed by a final extension at 72 uC for 7 minutes [8].

The M-strains are defined in this paper as *M. tuberculosis *strains which have a matched IS*6110*-RFLP band pattern including those with one band difference, if any (Figure 1); are resistant to Streptomycin; have any of the following VNTR profiles, either M4-substrain with four tandem repeat copies at the ETR C locus, i.e., 223315173533-4243-8844433-4337, or M5-substrain with five tandem repeat copies at the same locus including one copy number difference at the VNTR4156 locus, i.e., either 223315173533-4253-8844433-4337 or 223315173533-4253-8844434-4337 (Table 1)".

**Table 1:** Patient and VNTR Profiles whose *M. tuberculosis *Indicated M-strains

No.	M-strain Type (*1)	Year Patient Registed	TB diagnosis (*2)	sex (*3)	Age (years old)	Internet-café use (*4)	Homeless (*5)	Drug Resistance (*6)	VNTR profiles (*7)
1	M5	2004	univ	M	22	No	No	SM	**223315173533-42 53-884443 4-4337**

2	M5	2004	univ	M	22	No	No	SM	**223315173533-42 53-884443 3-4337**

3	M5	2004	univ	M	22	No	No	SM	**223315173533-42 53-884443 4-4337**

4	M5	2004	univ	M	22	Yes	No	SM	**223315173533-42 53-884443 4-4337**

5	M5	2004	univ	F	21	No	No	SM	**223315173533-42 53-884443 3-4337**

6	M5	2004	univ	M	22	No	No	SM	**223315173533-42 53-884443 4-4337**

7	M5	2004	univ	M	22	No	No	SM	**223315173533-42 53-884443 3-4337**

8	M5	2004	univ	M	22	No	No	SM	**223315173533-42 53-884443 3-4337**

9	M5	2004	univ	M	22	No	No	SM & INH	**223315173533-42 53-884443 4-4337**

10	M5	2004	univ	M	22	No	No	SM & INH	**223315173533-42 53-884443 4-4337**

11	M5	2004	univ	M	22	No	No	SM	**223315173533-42 53-88 2443 4-4337**

12	M5	2005	univ	F	unknown	No	No	SM	**223315173533-42 53-884443 4-4337**

13	M5	2005	univ	M	22	No	No	SM	**223315173533-42 53-884443 4-4337**

14	M5	2005	univ	M	24	No	No	SM	**223315173533-42 53-884443 3-4337**

15	M5	2005	univ	M	21	No	No	SM	**223315173533-42 53-884443 4-4337**

16	M5	2005	univ	M	22	No	No	SM	**223315173533-42 53-884443 4-4337**

17	M5	2005	univ	M	23	No	No	SM	**223315173533-42 53-884443 3-4337**

18	M5	2005	univ	M	22	No	No	SM	**223315173533-42 53-884443 4-4337**

19	M5	2006	s	M	58	No	Yes	SM	**223315173533-42 53-884443 3-4337**

20	M5	2007	s	M	39	Yes	Yes	SM	**223315173533-42 53-884443 3-4337**

21	M4	2003	s	M	59	No	No	SM	**223315173533-42 43-884443 3-4337**

22	M4	2004	univ	M	22	Yes	No	SM	**223315173533-42 43-884443 3-4337**

23	M4	2004	univ	M	24	No	No	SM	**223315173533-42 43-884443 3-4337**

24	M4	2004	Ob	M	24	No	No	SM	**223315173533-42 43-884443 3-4337**

25	M4	2004	Ob	M	35	No	No	SM	**223315173533-42 43-884443 3-4337**

26	M4	2004	ka	M	49	No	Yes	SM	**223315173533-42 43-884443 3-4337**

27	M4	2004	ka	M	31	No	No	SM	**223315173533-42 43-884443 3-4337**

28	M4	2004	ka	M	41	No	No	SM	**223315173533-42 43-884443 3-4337**

29	M4	2004	s	M	65	Yes	Yes	SM	**223315173533-42 43-884443 3-4337**

30	M4	2005	Ob(ka)	M	47	Yes	Yes	SM	**223315173533-42 43-884443 3-4337**

31	M4	2005	Ob(s)	M	28	Yes	No	SM	**223315173533-42 43-884443 3-4337**

32	M4	2005	Ob	M	31	Yes	Yes	SM	**223315173533-42 43-884443 3-4337**

33	M4	2005	Ob	M	25	Yes	Yes	SM	**223315173533-42 43-884443 3-4337**

34	M4	2005	ka	M	30	No	No	SM	**223315173533-42 43-884443 3-4337**

35	M4	2005	ka	F	56	No	No	SM	**223315173533-42 43-884443 3-4337**

36	M4	2005	ka	M	45	Yes	Yes	SM	**223315173533-42 43-884443 3-4337**

37	M4	2006	ka	M	67	No	No	SM	**223315173533-42 43-884443 3-4337**

38	M4	2006	ka	M	58	No	No	SM	**223315173533-42 43-884443 3-4337**

39	M4	2006	s	F	33	No	No	SM	**223315173533-42 43-884443 3-4337**

40	M4	2007	ka	M	46	No	No	SM	**223315173533-42 43-884443 3-4337**

41	M4	2007	ka	M	54	No	No	SM	**223315173533-42 43-884443 3-4337**

42	M4	2007	ka	M	44	No	No	SM	**223315173533-42 43-684443 3-4337**

43	M4	2007	ka	F	39	No	No	SM	**223315173533-42 43-884443 3-4337**

44	M4	2007	ka	M	55	No	No	SM	**223315173533-42 43-884443 3-4337**

45	M4	2007	s	M	50	Yes	Yes	SM	**223315173533-42 43-884443 3-4337**

46	M4	2007	s	M	45	No	Yes	SM	**223315173533-42 43-884443 3-4337**

*1: "M4" indicates the M4-substrain with four tandem repeat copies at the ETR C locus. "M5" indicates the M5-substrain with five tandem repeat copies at the ETR C locus.

*2: "univ" indicates a TB patient detected through the contact investigation related to the tuberculosis outbreak at the university campus mentioned in the text.

"Ob" indicates a TB patient detected through contact investigations related to tuberculosis outbreak incidents other than the one at the university campus mentioned above.

"s" indicates a TB patient detected through the population-based DNA fingerprinting surveillance of *M. tuberculosis *in Shinjuku City.

"ka" indicates a TB patient detected through the hospital-based DNA fingerprinting surveillance of *M. tuberculosis *in Kawasaki City.

*3: "M" indicates male and "F" indicates female

*4: "Yes" indicates a TB patient who claimed to have a past history of the use of some internet cafés in the TMA within the past two years.

*5: A homeless person is defined as a person whose legal address is unknown or unstable at least within the past two years.

*6: Anti-tuberculosis agents the isolated *M. tuberculosis *indicated resistance. "SM" stands for Streptomycin, "INH" stands for Isoniazid.

*7: The 12 digits at the first part of the VNTR profile indicate the tandem repeat copy numbers according to the following loci set order: MIRU2, MIRU4, MIRU10, MIRU16, MIRU20, MIRU23, MIRU24, MIRU26, MIRU27, MIRU31, MIRU39, MIRU40 (Reference 5).

The 4 digits at the second part of the VNTR profile indicate the tandem repeat copy numbers according to the following loci set order: ETR A, ETR B, ETR C, ETR F (Reference 6).

The 7 digits at the third part of the VNTR profile indicate the tandem repeat copy numbers according to the following loci set order: QUB11b, QUB26, Mtub04, Mtub21, Mtub30, Mtub39, VNTR4156 (Reference 7).

The 4 digits at the last part of the VNTR profile indicate the tandem repeat copy numbers according to the following loci set order: QUB15, Mtub24, VNTR2372, VNTR3336 (Reference 8).

The epidemiological linkage, among those from whom M-strain was isolated, was investigated through routine or outbreak-related contact investigations by the PHC staff or by the infection control team at the above-mentioned university. The investigation was made mainly through interviews with the patients by the staff mentioned above. Most of the interviews had been done before the results of the molecular analyses became available.

An internet-café described in this paper is in general a type of cafeteria, being popular among youngsters for passing time and having a rest, which is commonly located in buildings close to a railway or an underground station in urban settings and is usually opened for 24 hours a day. At the place, they can spend some time by using a computer with Internet access. It is also commonly used as a temporary residence for homeless people to stay during night as its price is relatively inexpensive at night [12].

According to the Ethical Guidelines for Epidemiological Research by the government of Japan, informed consent does not necessarily need to be obtained from research subjects to conduct an observational study using the existing epidemiological data if the study does not use any human biological specimens [13]. Therefore informed consent was not obtained for this study because the present study was an observational study using anonymous and unlinked data of TB patients, whose data sources were existing three databases that had already been collected and analyzed. The informed consent, however, had been routinely verbally obtained regarding the DNA fingerprinting surveillance of *M. tuberculosis *from each of the bacillary positive tuberculosis patients registered in the Shinjuku PHC by the PHC staff, and the documented consent forms were also routinely obtained from each of the TB patients by the hospital staff in Kawasaki City. The study protocol was approved by the Institutional Review Board Committee of the Research Institute of Tuberculosis with the reference number 21-1.

Chi-square test was applied for the comparison between two sample proportions and Student's t test was applied for the comparison between two sample means. The p-value of 0.05 was set as a statistically significant level.

## Results

Out of a total of 664 isolates collected during the study period; 464 isolates from the TB patients registered in Shinjuku City, 176 isolates from those in Kawasaki City, 20 isolates from those related to the outbreak at a university campus in Yokohama City, and the other four isolates from those related to the outbreak other than the university, 57 (57/664 = 8.6%) were detected to have a matched IS*6110*-RFLP band pattern including one band difference, if any. Among them, 55 isolates (55/57 = 96.5%) showed resistance to Streptomycin. The other two isolates were pan-susceptible to the four major anti-tuberculosis agents including Streptomycin. Among these 55 isolates, nine isolates were excluded from the analysis because their VNTR profiles significantly differed (seven strains showed VNTR profile as 223215173433-4243-8844433-4337, one strain as 223315173533-4244-8844433-4337, and the other as 213315173533-4243-8844433-4337). Consequently 46 isolates of *M. tuberculosis *(46/664 = 6.9%) were identified as M-strains, showing an identical or a very similar VNTR profile. All of them belonged to Beijing-genotype strains by the spoligotyping analysis (14, data not shown). Among them, only two isolates were resistant to Isoniazid as well (No.9 and 10 in Table 1). There was no significant difference in the proportion of gender distribution between the total 664 patients and the 46 patients with M-strains (78.2% vs. 89.1%, Chi-square = 3.11, p = 0.780), while the mean age of the total patients was significantly higher than that of the patients with M-strains (53.6 years old vs. 35.0 years old, Student's t = 4.527, p = 0.000).

These 46 isolates were further divided into two groups based on the VNTR analysis on the ETR-C locus. 26 M-strains had four copies of tandem repeat at the ETR-C locus (No.21 through 46), designated as M4-substrains, i.e., 223315173533-4243-8844433-4337. Among these M4-substrains, only one isolate showed six copies of tandem repeat at the QUB11b locus (No.42). The other 20 M-strains, which have five copies of tandem repeat at the ETR-C locus, designated as M5-substrains, i.e., either 223315173533-4253-8844433-4337 or 223315173533-4253-8844434-4337, eight isolates showed three copies and 12 isolates showed four copies of tandem repeat at the VNTR4156 locus. 18 isolates (No.1 through No.18) were obtained from the patients related to the outbreak-related contact investigations at the university campus in Yokohama City. It was therefore suspected that all of these 18 patients had been infected from the same index source related to the outbreak at the university (No.1). While the remaining two M5-substrains (No.19, 20), both of them detected from homeless people, were identified through the population-based DNA fingerprinting surveillance of *M. tuberculosis *in Shinjuku City, the epidemiological linkage of these two patients with other patients with M5-substrains was not confirmed.

Alternatively, among the 26 M4-substrains, two isolates (No.22, 23) were obtained from the patients concerning the above-mentioned outbreak of TB at the university campus, four isolates (No.24, 25, 32, 33) were from the other outbreak-related contacts in the TMA, six isolates (No.21, 29, 31, 39, 45, 46) were through the population-based DNA fingerprinting surveillance of *M. tuberculosis *in Shinjuku City, and the remaining 14 isolates were through the hospital-based DNA fingerprinting surveillance of *M. tuberculosis *in Kawasaki City. Epidemiological linkage among the patients with M4-substrains was confirmed only between No.30 and No.31 patients. The No.31 patient, who was 28 years old when he contracted TB, had been working at the front desk of an internet-café in the TMA where the No.30 patient, who was 47 years old homeless, stayed quite a long period, and was coughing whilst he was there. It was therefore confirmed that the No.30 patient was the source of infection to the No.31 patient. Both of the No.22 and No.23 patients were detected through the contact investigation related to the above-mentioned university outbreak of TB in Yokohama City but their TB strains exceptionally belonged to the M4-substrain. The No.22 patient had worked at an internet-café before he contracted TB and had no contact with the index case (No.1). Therefore it was suspected that the infection source of the No.22 patient might have been different from the index case. On the other hand, the infectious source of the No.23 patient was not confirmed to be different from the index case.

There was a tendency where patients whose *M. tuberculosis *isolates belonged to M4-substrains had higher proportion to have paid a visit to some internet-cafés in the TMA within the past two years prior to the TB registration than those whose *M. tuberculosis *isolates belonged to M5-substrains, although it is not statistically significant (30.8% vs. 10.0%, Exact p = 0.150).

## Discussion

The M-strains were sub-divided into M4-substrains and M5-substrains according to the copy number difference at the ETR C locus. The copy number at this locus showed relatively low diversity [7], furthermore its diversity was lower among Beijing-genotype strains than among non-Beijing-genotype strains [8]. The sub-grouping of M-strains, M4-substrains and M5-substrains, according to the difference of tandem repeat copy numbers at the ETR C locus was supported by the epidemiological investigation results as described above.

As for M5-substrains, they were further divided into two strain groups, one was a group with three tandem repeat copies and the other with four copies at the VNTR4156 locus. The Hunter-Gaston Diversity Index at the VNTR4156 locus was relatively low among Beijing-genotype strains isolated in Japan as reported elsewhere [8]. While some patients had very close contact with the index case (No.1) and some of them were acknowledged as members of the definite contact group with the index case, we could not epidemiologically differentiate the patients into two groups according to the copy number at the VNTR4156 locus. It was suggested therefore that one copy number difference at the VNTR4156 locus of M5-substrains had occurred by chance at some point on their transmission process.

Concerning M4-substrains, although it was not confirmed by patient interviews, it was suggested that transmission of the M4-substrains, to some extent, has a possible connection to the utilization of internet-cafés. The data of the population-based DNA fingerprinting surveillance of *M. tuberculosis *in Shinjuku City, which collected around 85% of all isolates of *M. tuberculosis *of bacillary positive tuberculosis patients city-wide, suggested that its prevalence might not be high enough to be considered as an endemic strain [1]. It showed that only eight out of 464 isolates (1.7%) belonged to M-strains in Shinjuku City. In addition, the data from a nationwide drug resistance survey of *M. tuberculosis *in Japan in 2002 suggested that thus far, M-strains have not spread widely and nationwide [15]. It showed that four among 325 randomly selected samples of *M. tuberculosis *from the survey indicated the matched IS*6110*-RFLP band pattern as that of M-strain, however none of them was identified as M-strain as described above (unpublished data).

The annual notification rate of bacillary TB patients in Japan has been declining from 14.6 in 1980 to 12.0 per 100 000 population in 2006. In accordance with the decline of the number of TB patients, the prevalence of TB infection was estimated to decline among all age groups especially among young age groups [16]. Meanwhile, TB patients have been geographically concentrated mainly in urban settings in Japan where there are a number of people such as homeless people who have higher chances to be infected with and develop TB. For instance, the annual notification rates of bacillary TB patients per 100 000 population in mega-cities like Tokyo Metropolitan (15.2), Osaka City (35.4), Nagoya City (20.0), and Kawasaki City (16.1) were far above the national average (12.0) in 2006 [17]. Considering the very low level of TB infection among young age groups, it is expected that a TB outbreak could be highly possible among young age groups once a contagious TB patient has a contact with them. For instance, Nakanishi *et al. *reported outbreak incidents of TB including homeless people and the workers at several public saunas in Shinjuku City where homeless patients often stayed with other homeless workers [18]. They pointed out the possibility of transmission of TB in such places where quite a few homeless people stay for a long time as temporary residence.

We reported relatively high genotype clustering rate among homeless people (unadjusted Odds Ratio: 3.21, 95% Confidence Intervals 1.98–5.21) in Shinjuku City and many genotype clusters were a mixture of homeless and non-homeless patients, which may indicate transmission between these two population segments [1]. In line with this, the present study suggested that specific Streptomycin resistant M4-substrains have been transmitting among both homeless people and contacts with them in internet-cafés. Internet-cafés in addition to public saunas are currently used as temporary residence for a considerable number of homeless people in urban areas in Japan. Furthermore, they are the sites where many youngsters stay concurrently. This implies that not only close contact among family members or friends but also casual contact like those who share a common floor or space in an internet-café could be a focus of transmission of TB. Bifani *et al. *reported a spread of multiple drug resistant *M. tuberculosis *widely across several states around New York City, and warned significant risk for TB control in the future [19]. The present study similarly showed a spread of Streptomycin resistant specific genotype tuberculosis strains in urban settings in Japan.

The epidemiological investigation in the context of the present study has been limited to those whose strains sharing the closely related genotype caused the outbreak in a university campus, therefore this limits the ability to draw conclusions clearly as to the specific risk factors for acquisition of this strain. Nevertheless, the results of current molecular as well as epidemiological analysis successfully described the tip of the iceberg regarding the spread of specific Streptomycin resistant *M. tuberculosis *strains in the urban context in the TMA. The M-strains, which are resistant to Streptomycin, caused an outbreak of TB at a university involving numerous young age group people and also spread significantly in the urban area. All of the 46 TB patients infected with M-strains were not chronic or treatment failure cases but initial cases, which implies that the M-strains successfully transmit among people and induce development of active TB.

## Conclusion

Although firm conclusions could not be reached through the present study, it suggested that we have to take into consideration that TB could transmit in congregated facilities like internet-cafés where TB high-risk people and general people share common spaces for relatively long time. The results of this study also imply that we further need to monitor the spread of this strain across the TMA and clarify the transmission routes precisely so that we will be able to control the transmission effectively in an urban context in Japan.

## Competing interests

The authors declare that they have no competing interests.

## Authors' contributions

All authors contributed to conduct the present study significantly. AO, MM, NH, GO, MN, HM, and MO collected data and analysed. YM and SM performed DNA fingerprinting analysis. AO wrote the manuscript and YM, NH, GO, KU, MO, NY, SK, TM, and NI edited it. All the 14 authors approved the contents of the manuscript.

## Pre-publication history

The pre-publication history for this paper can be accessed here:

http://www.biomedcentral.com/1471-2334/9/138/prepub
