# Correction: Transmission of Specific Genotype Streptomycin Resistant Strains of *Mycobacterium tuberculosis *in the Tokyo Metropolitan Area in Japan

**DOI:** 10.1186/1471-2334-10-79

**Published:** 2010-03-29

**Authors:** Akihiro Ohkado, Yoshiro Murase, Masaaki Mori, Naoki Hasegawa, Goro Otsuka, Michiko Nagamine, Hideo Maeda, Kazuhiro Uchimura, Masako Ohmori, Norio Yamada, Shinji Maeda, Seiya Kato, Toru Mori, Nobukatsu Ishikawa

**Affiliations:** 1 Department of Epidemiology and Clinical Research, Research Institute of Tuberculosis, Japan Anti-Tuberculosis Association, Matsuyama 3-1-24, Kiyose, Tokyo, Japan; 2 Department of Reference for Mycobacteria, Research Institute of Tuberculosis, Japan Anti-Tuberculosis Association, Kiyose, Tokyo, Japan; 3 Health Centre, Keio University, Yokohama, Japan; 4 Department of Respiratory Medicine, Keio University, Yokohama, Japan; 5 Department of Health and Clinical Medicine, Bureau of Health and Welfare, Kawasaki, Japan; 6 Bureau of Social Welfare and Public Health, Tokyo Metropolitan Government, Shinjuku, Tokyo, Japan; 7 Department of International Cooperation, Research Institute of Tuberculosis, Japan Anti-Tuberculosis Association, Kiyose, Tokyo, Japan; 8 Research Institute of Tuberculosis, Japan Anti-Tuberculosis Association, Kiyose, Tokyo, Japan

## Correction

We noticed a one-digit error on the tandem repeat number at the MIRU 20 after the publication of our article [1]. This was due to a typing error. The tandem repeat number at the MIRU 20 should be 2 instead of 1. All the figures indicating the tandem repeat number at the MIRU 20, i.e. the fifth digit of the VNTR profile from the left end of the set of digits, should be corrected in the text and correct information is provided in the revised Table 1. The first 12 digits of the VNTR profiles in the Methods part and Results part as well as in the Table 1 should read as 223325173533- instead of 223315173533- (Table 1).

**Table 1:** Patient and VNTR Profiles whose *M.tuberculosis *Indicated M-strains

**No**.	M-strain Type (*1)	Year Patient Registered	TB Diagnosis (*2)	Sex (*3)	Age (years old)	Internet-café Use (*4)	Homeless (*5)	Drug Resistance (*6)	VNTR profiles (*7)
1	M5	2004	univ	M	22	No	No	SM	**223325173533-42****5****3-884443****4****-4337**

2	M5	2004	univ	M	22	No	No	SM	**223325173533-42****5****3-884443****3****-4337**

3	M5	2004	univ	M	22	No	No	SM	**223325173533-42****5****3-884443****4****-4337**

4	M5	2004	univ	M	22	Yes	No	SM	**223325173533-42****5****3-884443****4****-4337**

5	M5	2004	univ	F	21	No	No	SM	**223325173533-42****5****3-884443****3****-4337**

6	M5	2004	univ	M	22	No	No	SM	**223325173533-42****5****3-884443****4****-4337**

7	M5	2004	univ	M	22	No	No	SM	**223325173533-42****5****3-884443****3****-4337**

8	M5	2004	univ	M	22	No	No	SM	**223325173533-42****5****3-884443****3****-4337**

9	M5	2004	univ	M	22	No	No	SM & INH	**223325173533-42****5****3-884443****4****-4337**

10	M5	2004	univ	M	22	No	No	SM & INH	**223325173533-42****5****3-884443****4****-4337**

11	M5	2004	univ	M	22	No	No	SM	**223325173533-42****5****3-88****2****443****4****-4337**

12	M5	2005	univ	F	unknown	No	No	SM	**223325173533-42****5****3-884443****4****-4337**

13	M5	2005	univ	M	22	No	No	SM	**223325173533-42****5****3-884443****4****-4337**

14	M5	2005	univ	M	24	No	No	SM	**223325173533-42****5****3-884443****3****-4337**

15	M5	2005	univ	M	21	No	No	SM	**223325173533-42****5****3-884443****4****-4337**

16	M5	2005	univ	M	22	No	No	SM	**223325173533-42****5****3-884443****4****-4337**

17	M5	2005	univ	M	23	No	No	SM	**223325173533-42****5****3-884443****3****-4337**

18	M5	2005	univ	M	22	No	No	SM	**223325173533-42****5****3-884443****4****-4337**

19	M5	2006	s	M	58	No	Yes	SM	**223325173533-42****5****3-884443****3****-4337**

20	M5	2007	s	M	39	Yes	Yes	SM	**223325173533-42****5****3-884443****3****-4337**

21	M4	2003	s	M	59	No	No	SM	**223325173533-42****4****3-884443****3****-4337**

22	M4	2004	univ	M	22	Yes	No	SM	**223325173533-42****4****3-884443****3****-4337**

23	M4	2004	univ	M	24	No	No	SM	**223325173533-42****4****3-884443****3****-4337**

24	M4	2004	Ob	M	24	No	No	SM	**223325173533-42****4****3-884443****3****-4337**

25	M4	2004	Ob	M	35	No	No	SM	**223325173533-42****4****3-884443****3****-4337**

26	M4	2004	ka	M	49	No	Yes	SM	**223325173533-42****4****3-884443****3****-4337**

27	M4	2004	ka	M	31	No	No	SM	**223325173533-42****4****3-884443****3****-4337**

28	M4	2004	ka	M	41	No	No	SM	**223325173533-42****4****3-884443****3****-4337**

29	M4	2004	s	M	65	Yes	Yes	SM	**223325173533-42****4****3-884443****3****-4337**

30	M4	2005	Ob(ka)	M	47	Yes	Yes	SM	**223325173533-42****4****3-884443****3****-4337**

31	M4	2005	Ob(s)	M	28	Yes	No	SM	**223325173533-42****4****3-884443****3****-4337**

32	M4	2005	Ob	M	31	Yes	Yes	SM	**223325173533-42****4****3-884443****3****-4337**

33	M4	2005	Ob	M	25	Yes	Yes	SM	**223325173533-42****4****3-884443****3****-4337**

34	M4	2005	ka	M	30	No	No	SM	**223325173533-42****4****3-884443****3****-4337**

35	M4	2005	ka	F	56	No	No	SM	**223325173533-42****4****3-884443****3****-4337**

36	M4	2005	ka	M	45	Yes	Yes	SM	**223325173533-42****4****3-884443****3****-4337**

37	M4	2006	ka	M	67	No	No	SM	**223325173533-42****4****3-884443****3****-4337**

38	M4	2006	ka	M	58	No	No	SM	**223325173533-42****4****3-884443****3****-4337**

39	M4	2006	s	F	33	No	No	SM	**223325173533-42****4****3-884443****3****-4337**

40	M4	2007	ka	M	46	No	No	SM	**223325173533-42****4****3-884443****3****-4337**

41	M4	2007	ka	M	54	No	No	SM	**223325173533-42****4****3-884443****3****-4337**

42	M4	2007	ka	M	44	No	No	SM	**223325173533-42****4****3-****6****84443****3****-4337**

43	M4	2007	ka	F	39	No	No	SM	**223325173533-42****4****3-884443****3****-4337**

44	M4	2007	ka	M	55	No	No	SM	**223325173533-42****4****3-884443****3****-4337**

45	M4	2007	s	M	50	Yes	Yes	SM	**223325173533-42****4****3-884443****3****-4337**

46	M4	2007	s	M	45	No	Yes	SM	**223325173533-42****4****3-884443****3****-4337**

*1: "M4" indicates the M4-substrain with four tandem repeat copies at the ETR C locus. "M5" indicates the M5-substrain with five tandem repeat copies at the ETR C locus.

*2: "univ" indicates a TB patient detected through the contact investigation related to the tuberculosis outbreak at the university campus mentioned in the text.

"Ob" indicates a TB patient detected through contact investigations related to tuberculosis outbreak incidents other than the one at the university campus mentioned above.

"s" indicates a TB patient detected through the population-based DNA fingerprinting surveillance of *M.tuberculosis *in Shinjuku City.

"ka" indicates a TB patient detected through the hospital-based DNA fingerprinting surveillance of *M.tuberculosis *in Kawasaki City.

*3: "M" indicates male and "F" indicates female.

*4: "Yes" indicates a TB patient who claimed to have a past history of the use of some internet cafés in the TMA within the past two years.

*5: A homeless person is defined as a person whose legal address is unknown or unstable at least within the past two years.

*6: Anti-tuberculosis agents the isolated *M.tuberculosis *indicated resistance. "SM" stands for Streptomycin, "INH" stands for Isoniazid.

*7: The 12 digits at the first part of the VNTR profile indicate the tandem repeat copy numbers according to the following loci set order: MIRU2, MIRU4, MIRU10, MIRU16, MIRU20, MIRU23, MIRU24, MIRU26, MIRU27, MIRU31, MIRU39, MIRU40 (Reference 5).

The 4 digits at the second part of the VNTR profile indicate the tandem repeat copy numbers according to the following loci set order: ETR A, ETR B, ETR C, ETR F (Reference 6).

The 7 digits at the third part of the VNTR profile indicate the tandem repeat copy numbers according to the following loci set order: QUB11b, QUB26, Mtub04, Mtub21, Mtub30, Mtub39, VNTR4156 (Reference 7).

The 4 digits at the last part of the VNTR profile indicate the tandem repeat copy numbers according to the following loci set order: QUB15, Mtub24, VNTR2372, VNTR3336 (Reference 8).

We do apologize to readers for any misunderstanding or inconvenience caused by this error.

## Pre-publication history

The pre-publication history for this paper can be accessed here:

http://www.biomedcentral.com/1471-2334/10/79/prepub
